# Etymologia: Bayesian Probability

**DOI:** 10.3201/eid2301.ET2301

**Published:** 2017-01

**Authors:** Ronnie Henry, Martin I. Meltzer

**Keywords:** etymologia, Bayesian probability, statistics, Thomas Bayes, theorem, prior events, current events

## Bayesian Probability

Thomas Bayes (1701–1761) (Figure) was a Presbyterian Minister, and how he become interested in statistics and probability is uncertain. Bayes presented his famous theorem on probability in “An Essay Towards Solving a Problem in the Doctrine of Chances,” which was published posthumously by his friend Richard Price in 1763. Bayes’s theorem provides a method of explicitly including prior events or knowledge when considering the probabilities of current events (for example, including a history of smoking when calculating the probability of developing lung cancer). Bayesian approaches use prior knowledge and information (e.g., probabilities) that may help reduce uncertainty in analysis and have therefore been increasingly adopted by analysts in public health.

**Figure:**
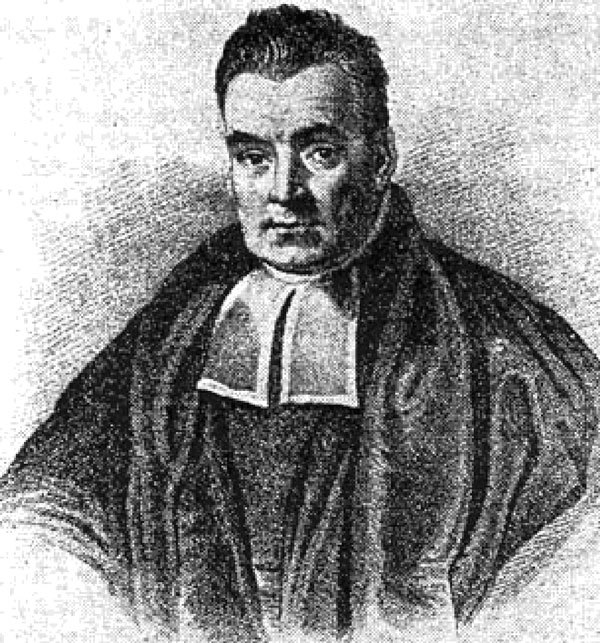
Thomas Bayes
